# Highly Reinforced Acrylic Resins for Hard Tissue Engineering and Their Suitability to Be Additively Manufactured through Nozzle-Based Photo-Printing

**DOI:** 10.3390/ma17010037

**Published:** 2023-12-21

**Authors:** Vito Gallicchio, Vincenzo Spinelli, Teresa Russo, Ciro Marino, Gianrico Spagnuolo, Carlo Rengo, Roberto De Santis

**Affiliations:** 1 Department of Neurosciences, Reproductive and Odontostomatological Sciences, University of Naples Federico II, Via S. Pansini 5, 80131 Naples, Italy; vito.gallicchio@unina.it (V.G.); vincenzo.spinelli@unina.it (V.S.); gspagnuo@unina.it (G.S.); 2 Institute of Polymers, Composites and Biomaterials, National Research Council of Italy, V.le J.F. Kennedy 54, Mostra d’Oltremare Pad. 20, 80125 Naples, Italy; teresa.russo@cnr.it; 3 University of Naples Federico II, P.le Tecchio 80, 80125 Naples, Italy; ciro.marino@unina.it; 4 Department of Prosthodontics and Dental Materials, University of Siena, 53100 Siena, Italy; carlorengo@alice.it

**Keywords:** particulate composites, light activated composites, mechanical analysis, additive manufacturing

## Abstract

Mineralized connective tissues represent the hardest materials of human tissues, and polymer based composite materials are widely used to restore damaged tissues. In particular, light activated resins and composites are generally considered as the most popular choice in the restorative dental practice. The first purpose of this study is to investigate novel highly reinforced light activated particulate dental composites. An innovative additive manufacturing technique, based on the extrusion of particle reinforced photo-polymers, has been recently developed for processing composites with a filler fraction (*w*/*w*) only up to 10%. The second purpose of this study is to explore the feasibility of 3D printing highly reinforced composites. A variety of composites based on 2,2-bis(acryloyloxymethyl)butyl acrylate and trimethylolpropane triacrylate reinforced with silica, titanium dioxide, and zirconia nanoparticles were designed and investigated through compression tests. The composite showing the highest mechanical properties was processed through the 3D bioplotter AK12 equipped with the Enfis Uno Air LED Engine. The composite showing the highest stiffness and strength was successfully processed through 3D printing, and a four-layer composite scaffold was realized. Mechanical properties of particulate composites can be tailored by modifying the type and amount of the filler fraction. It is possible to process highly reinforced photopolymerizable composite materials using additive manufacturing technologies consisting of 3D fiber deposition through extrusion in conjunction with photo-polymerization.

## 1. Introduction

In the biomedical field, advances in technologies have pushed research towards the fabrication of novel 3D structures for the repair or generation of hard and soft tissues, as well as for the design of advanced hydrogel-based devices for drug delivery [1,2,3,4,5,6,7].

**Figure 1:**
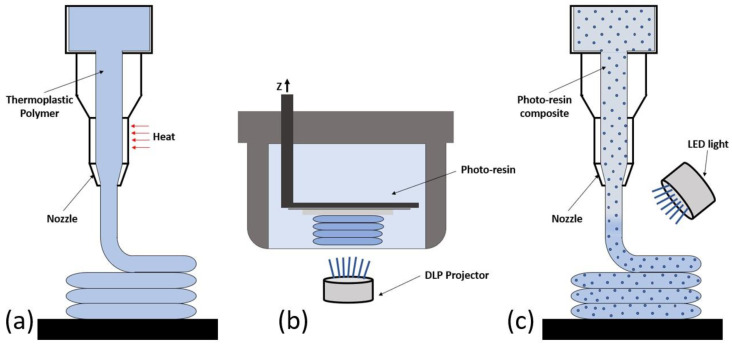
Additive manufacturing of composite materials: (**a**) fused deposition modeling; (**b**) stereolithography; (**c**) extrusion of particle reinforced photo-polymers.

Mineralized connective tissues such as spongy bone, compact bone, dentin, and enamel represent the hardest materials of human tissues [8,9]. Although it is recognized that continuous fiber reinforced polymers represent the best solution for mechanically mimicking mineralized tissues [10,11], unfortunately, additive manufacturing of continuous fiber reinforced polymers still shows limitations, thus encouraging the use of particle reinforced resins [12]. On the other hand, a high particle fraction is required to reinforce polymers in order to mimic mechanical properties of mineralized tissues [13,14], but the resulting high viscosity prevents 3D printing of highly filled particulate composites through traditional fused deposition modeling (Figure 1a) or through stereolithography (Figure 1b) [15,16]. However, an innovative additive manufacturing technology, based on the extrusion of particle reinforced photo-polymers, has been recently developed (Figure 1c) [17,18]. Although this technique has been explored for additively manufacturing composites with a filler fraction only up to 10%, this approach will be shown to be suitable for 3D printing highly reinforced composites.

The continuous growth and improvement of additive manufacturing technologies has further catalyzed the exploration of these materials and its processability allowing the precise layer-by-layer fabrication of complex geometries with improved mechanical properties and structural features [19]. Das et al., in recent years, stated how various additive manufacturing technologies were developed and applied to the biomedical field to obtain patient-specific medical devices such as implants and scaffolds, orthoses and prostheses or even drug delivery systems [20].

The chance offered to engineer materials with enhanced mechanical properties and tailored functionalities has been possible thanks to the integration of innovative micro- and nanofillers for reinforcing polymer matrices [21,22]. The advantages of combining polymer matrices and micro/nanoscale reinforcements to obtain resin-based composites have received significant attention for their applications in different industries ranging from biomedical devices to aerospace [23]. In particular, highly reinforced photo-curing resins have gained great attention in the field of restorative dentistry. As a result, within more than half a century of dental material developments, novel micro- and nanoreinforced methacrylate is commercially available for restoring damaged dentin and enamel [24,25,26].

Historically, polymer-based restorative materials have gained popularity for aesthetic reasons [27], and bisphenol-A glycidyl methacrylate (Bis-GMA), developed by Bowen in 1962 [28], in conjunction with diluents such as triethylene glycol dimethacrylate (TEGDMA) represents the most common polymeric matrix for dental restoration. From a biological perspective, the main drawback of in situ polymerized methacrilates is cytotoxicity ascribed to the amount of unreacted monomers [29,30]. However, for ex situ application (e.g., composite inlays/onlays, scaffolds, 3D printing, etc.), a significant enhancement of biocompatibility is observed by employing a postcure heat treatment [31]. From a mechanical perspective, the organic matrix alone is not capable of reproducing the mechanical performance of the mineralized tissues of a tooth. Accordingly, a variety of inorganic powders have been developed during the past half century in order to improve the stiffness and strength of restorative dental materials. At the beginning of their clinical use, particulate dental composites have been the choice for restoring anterior teeth; the color of the polymeric matrix can be properly changed by dispersing pigments, thus satisfying the patient’s aesthetic needs [32]. As microparticles for dental restorative materials have been developed, microfilled dental composites changed the scenario of restorative dentistry, and these composites, in conjunction with the etching technique developed by Buonocore [33], replaced the amalgam approach for restoring posterior teeth [34]. Novel nanoparticles for dental materials have been developed through nanotechnology. Accordingly, a new class of particulate composites, namely, hybrid composites and nanofilled composites [35], has been introduced in the market. The rule of mixtures of particulate composites [36] allows one to exhaustively recognize the effect of nanoparticles on the stiffness of particulate composites. While the stiffness of fiber reinforced composites is proportional to the amount of fibers [37], the stiffness of particulate composites is not proportional to the amount of particles, and this mechanical property can be increased only by using a high fraction value of the reinforcement phase. Nanoparticles allow filling the spaces among microparticles, thus boosting the effect of the reinforcement phase on the Young’s modulus according to the rule of mixtures. Therefore, nanoparticles increase the reinforcement loading efficiency.

This study aims to contribute to the ongoing investigation of resin-based micro- and nanocomposites by studying their mechanical properties and performing compressive analysis, which gives crucial indications for structural applications. By understanding and optimizing composite material characteristics, its potential can be exploited for a wide range of additive manufacturing applications, including the development of lightweight and durable components.

Novel formulations of micro- and nano-resin-based composites with different reinforcement types and concentrations were characterized in this study. The selection of the optimal filler and its dispersion into the polymer matrix play a key role in obtaining the desired mechanical properties of the composites for its application [38].

Two main objectives for this research were defined: First, to determine and understand the mechanical behavior of innovative formulations of resin-based micro- and nanocomposites. Second, to explore their potential for additive manufacturing applied to the biomedical field.

In this way, it is possible to fill the gap between cutting-edge material design and the design and production of functional components through additive manufacturing. By providing a better comprehension of mechanical performances of these materials, this study paves the way for future advancements and applications for the additive manufacturing industry.

## 2. Materials and Methods

Mechanical properties were evaluated through the compression test for composite materials. New formulations of micro- and nanocomposites were produced and provided by Kerr Dental Italia (Scafati, SA, Italy). Material compositions are depicted in Table 1.

Composite materials were divided into two groups: Group 1 includes all the basic formulations in which the total resin percentage and filler (TiO_2_) content varies. In Group 2, the total resin matrix, filler (TiO_2_) content, and the prepolymerized premix microparticle percentages are the same. Samples of Group 2 differ in their zirconium oxide nanoparticle amounts.

### 2.1. Compression Test

**Figure 2:**
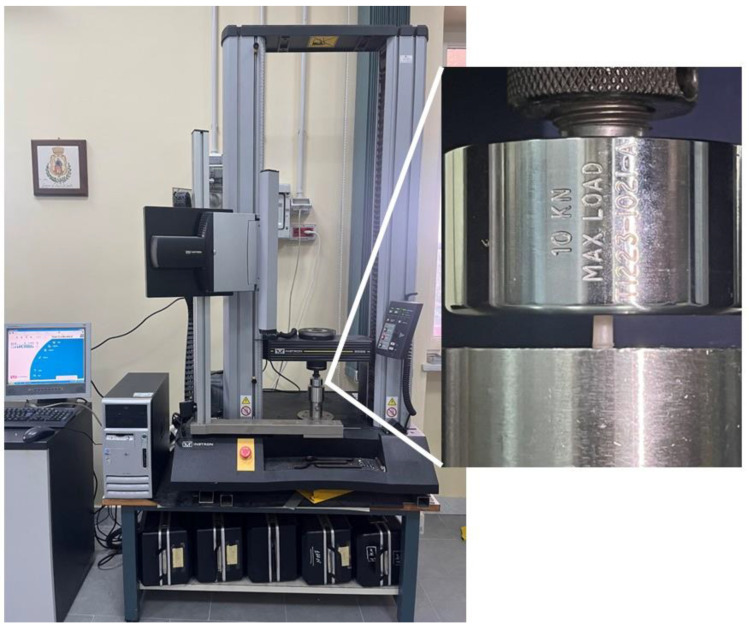
Compression test set-up.

Compressive properties of resin-based composites were investigated through the compression test (Figure 2).

#### 2.1.1. Specimen Preparation

Five specimens for each type of composite were prepared with a cylindrical Teflon mould (3 mm diameter, 4 mm height). Composite materials were injected into the cylindrical cavity of the mould and the bases were shut by microscope slides to prevent material flow and avoid a potential oxidation of the composite during curing. A 3 kg weight was positioned on the upper base of the mould for 1 min to stabilize the specimen shape.

Polymerization was achieved with the commercial LED source Ivoclar Power Phase. “H mode” of the LED source was used, obtaining a power output of 1200 mW/cm^2^, a wavelength of 385–515 nm, and an exposing time of 20 s. After polymerization, each specimen was conditioned by storing it for 3 days in a dark environment at room temperature.

#### 2.1.2. Compression Test Set-Up

Compression tests were performed with the Instron 5566 dynamometer (Instron Ltd., High Wycombe, UK) equipped with a 5 kN load cell (Figure 2) at a speed of 1 mm/min.

Preliminary, the dynamometer compliance was evaluated by running a compression test without the specimen. The load–displacement curve is characterized by a linear trend, and the dynamometer compliance (C) was deducted from the curve slope. The dynamometer compliance value, experimentally measured, was considered to evaluate the effective deformation of short specimens in compression.

Each specimen was then positioned between the dynamometer compression plates, and a 30 N preloading was used. The test was performed at a speed of 1 mm/min, and the load–displacement curve was recorded.

Stress vs. strain curves were estimated through the equations:

$$\sigma =\frac{F}{A};\varepsilon =\frac{∆l^{*}}{l_{0}}$$

where F is the applied force in N, A is the base area of the cylindrical specimen, *l*_0_ is the height of the specimen in mm, and Δl* is the shortening of the specimen considering the dynamometer compliance (C):

$$∆l^{*}=∆l-CF$$

where Δ*l* is the lowering of the dynamometer crosshead in mm.

The ratio of the maximum applied force and the cross-section area of the specimen was considered to evaluate mechanical strength. The ratio of maximum displacement and specimen height were evaluated to determine the strain to failure, while the steepness of the stress–strain curve in the elastic region depicts the Young’s modulus.

Finally, data were statistically analyzed using one-way ANOVA followed by Tukey’s test at a critical value of 0.05. The mean and standard deviation (SD) of compression strength, Young’s modulus, tangent modulus, and elongation at break for each formulation were calculated.

### 2.2. Additive Manufacturing of Composites

**Figure 3:**
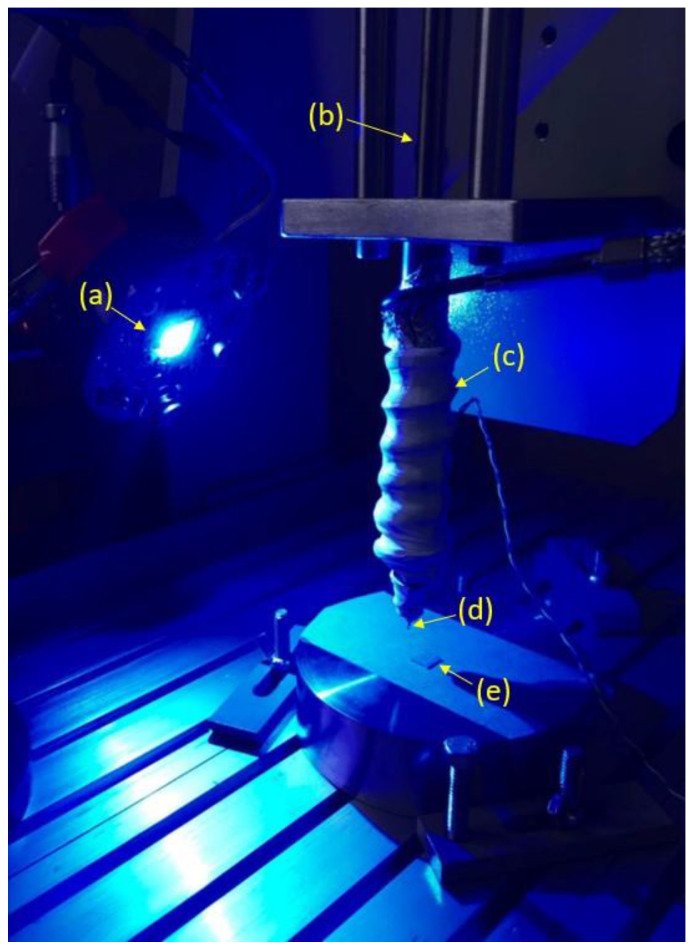
Setup of the nozzle-based 3D photo-printing equipment. (a) LED light; (b) actuator; (c) extruder; (d) needle; (e) four-layer 3D Scaffold.

The processability of the formulation that achieved the best mechanical features was tested. A 3D bioplotter AK12 (Naples, Italy) equipped with a Enfis Uno Air LED Engine (Swansea, UK) was used (Figure 3). The output power of the LED Engine was 4680 mW at a wavelength of 465 nm. Composite materials were extruded through a nozzle (Diameter of 800 µm) using a pressure of 10 bar at room temperature. Printing speed was set at 50 mm/min.

Four layers of photopolymerized composite scaffolds with a lay-down pattern of 0°/90° and a strand distance of 1500 µm were realized. Scaffolds had final dimension of 15.0 × 15.0 × 3.0 mm^3^. These scaffolds underwent a postcure heat treatment at 50 °C for 10 min.

## 3. Results and Discussions

**Figure 4:**
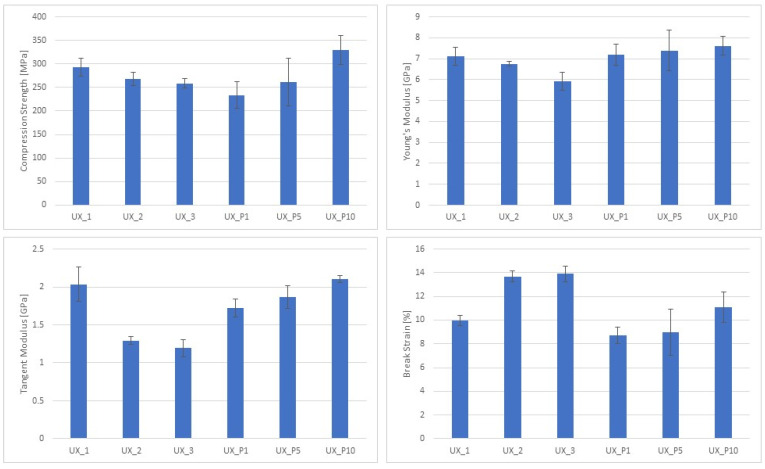
Effect of formulation variation and nanotechnology on the mechanical properties of Ultra XRV series composite materials.

Figure 4 reports the effect of the formulation variation and the effect of micro/nanometric particles on the mechanical properties (e.g., compression strength, Young’s modulus, tangent modulus, and displacement at break) of the Herculite XRV Ultra modified composite materials. Stress vs. strain curves can be found in the Supplementary Files. The use of inorganic nanoparticles for increasing mechanical properties of restorative resins has gained great attention in the last decade, and compressive properties are of paramount importance because highly mineralized tissues in the oral cavity (i.e., dentin and bone) mainly undergo a compressive state of stress during mastication; crack initiation at restorative sites causes secondary caries [26,39,40,41].

The results show interesting aspects of the various investigated composite materials tested in compression. In terms of formulation modification, by varying the resin/filler ratio, different properties are observed. Regarding Group 1, UX_1 that has a higher filler content (TiO_2_ = 64%) and a reduced amount of total resin percentage (Resin 1 = 18%, Resin 2 = 13%), compared to other basic formulations, has shown a great improvement. This formulation presents significant best values (*p* < 0.05) for compression strength (293.00 ± 18.47 MPa) and Young’s modulus (7.12 ± 0.41 GPa) compared only to UX_3 formulation. The tangent modulus achieves significant higher values (2.04 ± 0.23 GPa) (*p* < 0.05) compared to UX_2 and UX_3. Instead, the break strain presents significantly lower values (9.98 ± 0.42 mm/mm %) (*p* < 0.05) only compared to UX_3. This effect can be ascribed to the reduced amount of resin matrix, thus resulting in a toughness reduction. These results are in agreement with those of Zakaria M.R. et al. (2009), Moezzyzadeh M. (2011), Gill R. et al. (2017), and Lemon et al. (2020) that compared the compressive strength of hybrid and nanocomposites including the Herculite XRV, produced by Kerr, having a similar composition to UX_1 [42,43,44,45]. This hybrid dental composite has a clinical history longer than two decades [46]; it has a degree of conversion higher than other hybrid dental composites [47], and modifications of Herculite XRV with hydroxyapatite and silver nanoparticles have been recently proposed [48]. The compression strength (293.00 ± 18.47 MPa) measured for UX_1 (Figure 4) is consistent with the compression strength (307.08 ± 20.17 MPa) observed by Zakaria M.R. et al. (2009) [42]. Lower compression strength values (275.75 ± 76.99 MPa) are reported by Moezzyzadeh M. (2012). The discrepancy may be ascribed to specimen conditioning for 48 h in distilled water at 37 °C; the absorbed water plasticizes the composite reducing its strength [43]. Lower strength values (274 ± 15 MPa) have been measured also by Gill R. (2017). Again, this lower strength value may be ascribed to specimen conditioning (aging for 10 days), but also to an unsuitable photocuring process leading to a low degree of conversion [44,47]. Although Herculite XRV shows lower water absorption compared to other hybrid composites, specimen conditioning in a severe humid environment for 10 days further decreases the compression strength [47].

On the other hand, UX_3 presents the lowest filler content (TiO_2_ = 62%) and an increased total amount of resin percentage (Resin 1 = 20%, Resin 2 = 13%), and achieves significant lower values of compression strength (258.25 ± 10.12 MPa) (*p* < 0.05) only compared to UX_1. The Young’s modulus (5.92 ± 0.44 GPa) achieves significantly low values (*p* < 0.05) compared to UX_1 and UX_2. The tangent modulus (1.19 ± 0.11 GPa) achieves significantly lower values (*p* < 0.05) compared only to UX_1. The break strain presents significant higher values (13.90 ± 0.67 mm/mm %) (*p* < 0.05) compared only to UX_1. This effect can be ascribed to the increased amount of resin matrix, resulting in a great toughness improvement. Consistent with these results, the UX_2 formulation, having an intermediate value of filler (TiO_2_) content and total amount of resin matrix, achieves values of compression strength (268.18 ± 14.69 MPa), Young’s modulus (6.75 ± 0.11 GPa), tangent modulus (1.29 ± 0.05 GPa), and break strain (13.68 ± 0.46 mm/mm %). These values are in between of those observed for the UX_1 and UX_3 formulations. By reducing the total amount of resin matrix and increasing the filler content (e.g., TiO_2_) percentage, it is possible to enhance the mechanical properties of the initial composite formulation, although a reduction in material toughness is achieved (Figure 4). This result corroborates the well-known effect of particulate reinforcement amount on polymer-based composites established in the previous century and described by the rule of mixtures [13,36]. More recent theoretical and numerical investigations have been proposed for accurately describing the effect of reinforcement amount on compressive properties of highly filled dental composites [49].

For Group 2, the UX_P10 formulation, having the higher percentage of zirconia nanoparticles, achieves significantly higher values (*p* < 0.05) of compression strength (328.89 ± 30.60 MPa) compared to UX_P1, but not statistically significant compared to UX_P5. The Young’s modulus (7.61 ± 0.44 GPa) achieves higher values that are not statistically significant compared to UX_P1 and UX_P5. The tangent modulus and break strain, 2.10 ± 0.05 GPa and 11.10 ± 1.29 mm/mm %, respectively, are significantly higher (*p* < 0.05) compared to UX_P1. Moreover, UX_P5, having an intermediate percentage of zirconium oxide nanoparticles compared to UX_P1 and UX_P10, presents values that are in between the two endpoints. However, no statistically significant difference is observed. These results suggest that the higher percentage of zirconia nanoparticles added into the composite material enhances its characteristics in terms of compression strength, Young’s modulus, and tangent modulus.

By comparing the two groups and the formulations that achieved the best results (UX_1 and UX_P10), it can be observed that the formulation that presents the zirconia addition achieves higher values. However, no statistically significant difference is observed for compression strength, Young’s modulus, and tangent modulus. Instead, the break strain is significantly lower for the zirconia group formulation (*p* < 0.05). This result suggests that the addition of zirconia improves the mechanical properties of the composite material. On the other hand, the compliance of the composite with zirconia reduces with the addition of inorganic nanoparticles. These results are consistent with other studies that found an improvement of the Young’s modulus related to the addition of zirconia nanoparticles into the composite material. Moreover, they found that zirconia nanoparticles make materials harder but less flexible, negatively affecting the break strain as in our study [50].

A large inconsistency of Young’s modulus measured in compression for highly filled dental composites is reported in the literature [39,40,51,52]. Young’s modulus is defined as the ratio between the stress and strain in the initial linear static region, and the modality by which the strain is measured largely affects the estimation of Young’s modulus. In fact, if the compressive strain is evaluated through the cross-head displacement of the dynamometer [39,40], significantly lower Young’s modulus values are assessed. Instead, if the strain is measured through local deformation sensors [41], then higher Young’s modulus values are estimated [42,51]. In other words, the compliance of the dynamometric instrument has a large influence on the computation of the Young’s modulus. Through our experiments, the compliance of the dynamometer has been considered and the measurements of the Young’s modulus spanned between (5.92 ± 0.44 GPa) and (7.61 ± 0.44 GPa) depending on the filler amount. The detected Young’s modulus values are consistent with the values of highly filled composites measured by Masouras et al. (2008) and Lourenço et al. (2019), reporting Young’s modulus values of (7.15 ± 0.36 GPa) and (7.50 ± 0.73), respectively [42,51].

Regarding tangent modulus values (Figure 4), although the bilinear stress vs. strain behavior in compression of highly filled dental composites is largely documented [39,41,52,53] and consistent with the observed behavior (see Supplementary Files), little attention has been given to the values of the tangent modulus occurring in the second linear stage of the stress vs. strain curves in compression. The tangent modulus value is of paramount importance for defining the elastic constitutive equation of the highly filled composite material to be used in finite element analysis simulations of a restored tooth or a medical device such as scaffolds and prostheses for mineralized tissue. This aspect will be one of the objects of our future investigations.

Finally, the measured break strain of the investigated highly filled composites (i.e., UX_P1, UX_P5 and UX_P10) reported in Figure 4 spans between (8.75 ± 0.69 mm/mm %) and (11.10 ± 1.29 mm/mm %). These values are consistent with those detected for highly filled composites by Gungor et al. [54].

Nanotechnology represents a breakthrough in the synthesis of advanced and biomimetic materials. The relevant interest in particulate nanocomposites relies on the extrudability feature. The composite formulation showing the best mechanical properties (UX_P10) was successfully processed using the nozzle-based 3D photo-printing approach. To the authors’ knowledge, this can be considered as the first attempt for additively manufacturing, through extrusion in conjunction with photopolymerization, a highly filled composite device suitable for mimicking mechanical properties of hard tissues. Further characterization (e.g., scanning electron micrography) of the additively manufactured composites should be carried out to demonstrate that the investigated composite material maintains its nanometric nature. However, recent investigation through scanning electron micrography of nanoparticles modified dental composites having similar matrix composition [48,52,53,54] suggest that this is the case.

**Figure 5:**
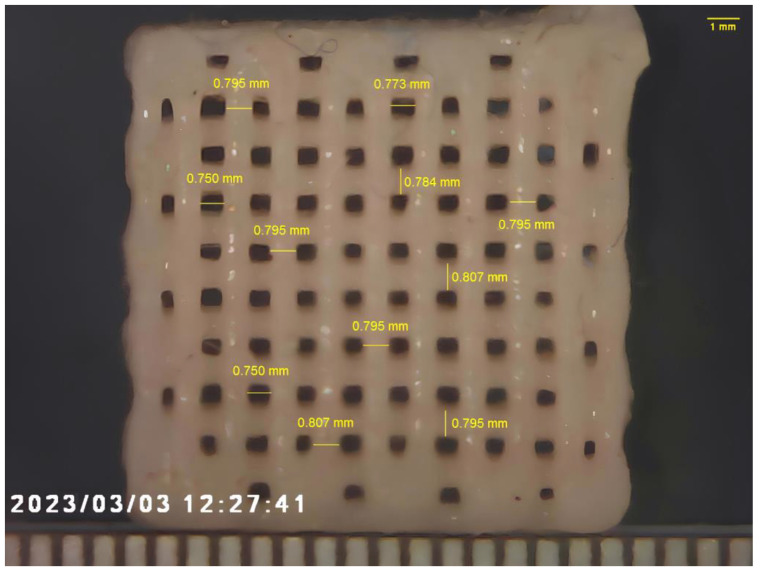
Four-layer 3D photo-printed nanocomposite scaffold using the UX_P10 formulation. The bottom scale bar is in mm.

Figure 5 shows the four-layer scaffold using the UX_P10 formulation. Pores are fully interconnected and the size of each pore is 750 µm (the difference between the strand distance and the fiber diameter). Although additive manufacturing based on the extrusion of particle reinforced photo-polymers has been recently developed [17,18], this technique has been explored for additively manufacturing composites with a filler fraction only up to 10%.

Recently, modified dental resins have been proposed as an alternative to poly(methyl-methacrylate)-based bone cements for overcoming the drawbacks of traditional bone cements, such as high shrinkage and high temperature levels, occurring after the exothermal free radical polymerization and unreacted monomer release, related to the low degree of conversion of bone cements [55,56]. Moreover, the stiffness of modified traditional bone cements using hydroxyapatite microparticles and TiO_2_ nanoparticles [57] spans between 1.51 ± 0.01 GPa and 4.76 ± 0.89 GPa, thus allowing one to reproduce only the stiffness of low-density trabecular bone of the mandible [9]. Instead, compression stiffness of modified highly filled dental composites (i.e., Young’s modulus in Figure 4), spanning between 5.92 ± 0.44 GPa and 7.61 ± 0.44 GPa, provide a good match with the compression stiffness of trabecular bone of human mandible spanning between 0.05 GPa and 16 Gpa according to bone density, mandible site, and direction [9]. Therefore, prompted by the above observations, we first explored the possibility to manufacture a highly filled composite scaffold through the additive manufacturing approach for bone tissue engineering. Future investigations will be devoted to additively manufacturing highly filled bone scaffolds in other districts of interest for the maxilla–facial surgery such as cranial bone defects. Nevertheless, there are dental fields that could benefit from the proposed technology. One of these fields could be the additive manufacturing of customized composite dental inlay/onlay. This possibility will be explored in the near future. Other fields of applications for the proposed technology in relation to dentistry that are worth exploring could be customized endodontic posts, bites, and orthodontic aligners.

The proposed technology, combining material design of highly reinforced composites and nozzle 3D photo-printing, may represent a significant breakthrough in restoring hard tissues. The speed of deposition at room temperature of highly filled composites represents the main limitation. However, it is possible to process these composite materials at temperature levels higher than room temperature, and a significant speed increase is expected. Thus, further investigations are needed.

Similar to fused deposition modeling (Figure 1a), one of the main limitations of the proposed nozzle 3D photo-printing is dimensional accuracy, and shrinkage is recognized to be the main source of dimensional error [58]. In fact, through fused deposition modeling, the printed material is deposited in the melt state, and a thermal shrinkage occurs as the material cools down at room temperature. The dimensional differences between the 3D model and the medical devices additively manufactured through fused deposition modeling may be as high as 2.67% [59]. Through nozzle 3D photo-printing, shrinkage occurs as a result of the photo-polymerization process, and a volumetric shrinkage of about 3.1% is expected for modified dental composites [56]. Nevertheless, a reduction of fiber diameter (modeled as 800 µm) can be observed after photo-polymerization of the scaffold, as reported in Figure 5. The diameter reduction represents a linear shrinkage, and it has been estimated to be about 0.7%; therefore, the volumetric shrinkage of fibers reported in Figure 5 may be estimated to be around 2.1% as a first approximation. However, further research involving the use of microtomography scans is needed for assessing the accuracy of the nozzle 3D photo-printing of highly filled scaffolds.

## 4. Conclusions

- It is possible to tailor mechanical properties of particulate composites by altering the total resin matrix percentage and the filler content.
- The stiffness and the strength of the composite material increase as the filler fraction increases, but a reduction of ductility (i.e., break strain) is observed as the filler fraction increases.
- Zirconium oxide nanoparticles allow one to achieve mechanical properties higher than those observed for the basic formulations.
- It is possible to process highly reinforced photopolymerizable composite materials using additive manufacturing technologies consisting of 3D fiber deposition through extrusion in conjunction with photo-polymerization. Further analyses will consist of manufacturing more components by varying process parameters, and by studying the results in terms of accuracy and structural behavior.

The current study paves the way for further formulation development in order to further improve mechanical properties of highly reinforced methacrylate suitable for 3D photo-printing. Future experiments are needed to investigate processability at different temperature levels of composite materials in order to increase the deposition speed, so that all of the advantages offered from this technology can be harnessed.

## Figures and Tables

**Table 1:** Material compositions.

	Material	Resin 1 (%) *	Resin 2 (%) **	Premix (%) ***	SiO_2_ (%)	TiO_2_ (%)	ZrO_2_ Nanoparticles (%)
Group 1	UX_1	18	13	-	5	64	-
	UX_2	20	12	-	5	63	-
	UX_3	20	13	-	5	62	-
Group 2	UX_P1	17	12	6	4	60	1
	UX_P5	16	11	6	4	58	5
	UX_P10	15	10	6	4	55	10

* Resin 1: 2,2-bis(acryloyloxymethyl)butyl acrylate; ** resin 2: trimethylolpropane triacrylate. *** Premix: pre-photopolymerized micro particles consisting of resin 1 and resin 2 in the ratio 5/4.

## Data Availability

Data are contained within the article and Supplementary Materials.

## Supplementary Materials

The following supporting information can be downloaded at: https://www.mdpi.com/article/10.3390/ma17010037/s1, Figure S1: Mechanical behavior of all the formulations of composite material. (a) Stress–strain curves; (b) elastic region; (c) second linear region.
