# Supplementary material for: Highly Reinforced Acrylic Resins for Hard Tissue Engineering and Their Suitability to Be Additively Manufactured through Nozzle-Based Photo-Printing
Source: Materials (Basel). 2023 Dec 21;17(1):37. doi: 10.3390/ma17010037 (PMC10779947; doi:10.3390/ma17010037)
Supplement: Supplementary file 1 [file materials-17-00037-s001.zip › materials-2735336-supplementary.pdf]

Figure S1 reports the mechanical behavior in compression for all the formulations of Ultra XRV. An initial portion detecting the elastic behavior of each composite's material formulation can be distinguished. The steepness of each curve in the linear region (dP/dy) was computed through the linear best fit. The behavior of all composite materials have in common a typical bi-linear model behavior. The first linear behavior, that affects the Young's modulus, is followed by a second linear behavior with a lower slope up to the break point. The bi-linear behavior of the material suggests to evaluate the tangent modulus to the second linear section (E2). A fragile break can be observed due to a vertical crash fracture, or a series of fractures before the definitive one.

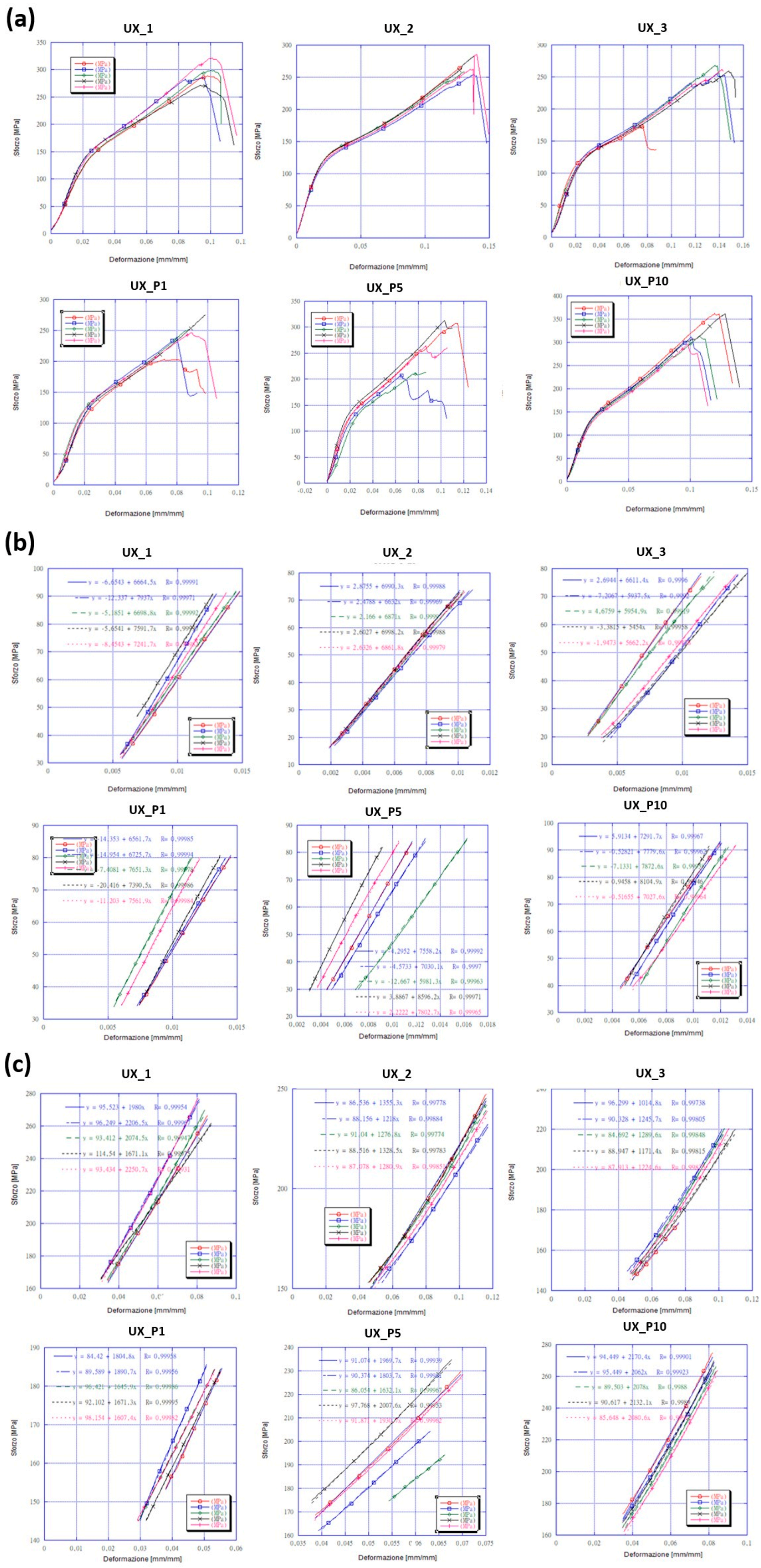

Figure S1. Mechanical behavior of all the formulations of composite material (a) Stress-strain curves; (b) Elastic region; (c) Second linear region.
